# Supplementary material for: Group 2 innate lymphoid cells are a non-redundant source of interleukin-5 required for development and function of murine B1 cells
Source: Nat Commun. 2024 Dec 4;15:10566. doi: 10.1038/s41467-024-54780-3 (PMC11618303; doi:10.1038/s41467-024-54780-3)
Supplement: Supplementary file 1 — Supplementary Information [file 41467_2024_54780_MOESM1_ESM.pdf]

Supplementary Figures 1-6

for

Group 2 innate lymphoid cells are an essential source of interleukin-5 required for development and function of murine B1 cells

Running title: Essential functions of ILC2s for B1 cell development

Karoline F. Troch[1,*], Manuel O. Jakob[1,*], Patrycja M. Forster[1], Katja J. Jarick[1], Jonathan Schreiber[2], Alexandra Preusser[1], Gabriela M. Guerra[3], Pawel Durek[3], Caroline Tizian[1], Nele Sterczyk[1], Sofia Helfrich[1], Claudia U. Duerr[1], David Voehringer[4], Mario Witkowski[1,5], David Artis[6,7,8,9,10], Tim Rollenske[2], Andrey A. Kruglov[3], Mir-Farzin Mashreghi[3,11], and Christoph S.N. Klose[1,#]

Authors' affiliations:

[1] Charité – Universitätsmedizin Berlin, corporate member of Freie Universität Berlin and Humboldt-Universität zu Berlin, Department of Microbiology, Infectious Diseases and Immunology, Hindenburgdamm 30, 12203 Berlin, Germany.

[2] Institute of Molecular Medicine and Experimental Immunology, University Hospital Bonn, Bonn, Germany.

[3] Deutsches Rheuma-Forschungszentrum (DRFZ), an Institute of the Leibniz Association, Berlin, Germany.

[4] Department of Infection Biology, University Hospital Erlangen and FAU Profile Center Immunomedicine (FAU I-MED), Friedrich-Alexander University Erlangen-Nuremberg, Erlangen, Germany

[5] The Picower Institute for Learning and Memory, Department of Brain and Cognitive Sciences, Massachusetts Institute of Technology, Cambridge, MA 02139, USA

[6] Jill Roberts Institute for Research in Inflammatory Bowel Disease, Weill Cornell Medicine, Cornell University, New York, NY 10021, USA

[7] Friedman Center for Nutrition and Inflammation, Weill Cornell Medicine, Cornell University, New York, NY 10021, USA.

[8] Joan and Sanford I. Weill Department of Medicine, Weill Cornell Medicine, Cornell University, New York, NY 10021, USA.

[9] Department of Microbiology and Immunology, Weill Cornell Medicine, Cornell University, New York, NY 10021, USA.

[10] Allen Discovery Center for Neuroimmune Interactions, Weill Cornell Medicine, Cornell University, New York, NY 10021, USA.

[11] German Center for Child and Adolescent Health (DZKJ), Partner Site Berlin, Berlin, Germany

* These authors contributed equally to the work

Corresponding author**:** Christoph S.N. Klose. Department of Microbiology, Infectious Diseases and Immunology Charité - Universitätsmedizin Berlin: Campus Benjamin Franklin
Hindenburgdamm 30 12203 Berlin, Germany. Email: christoph.klose@charite.de

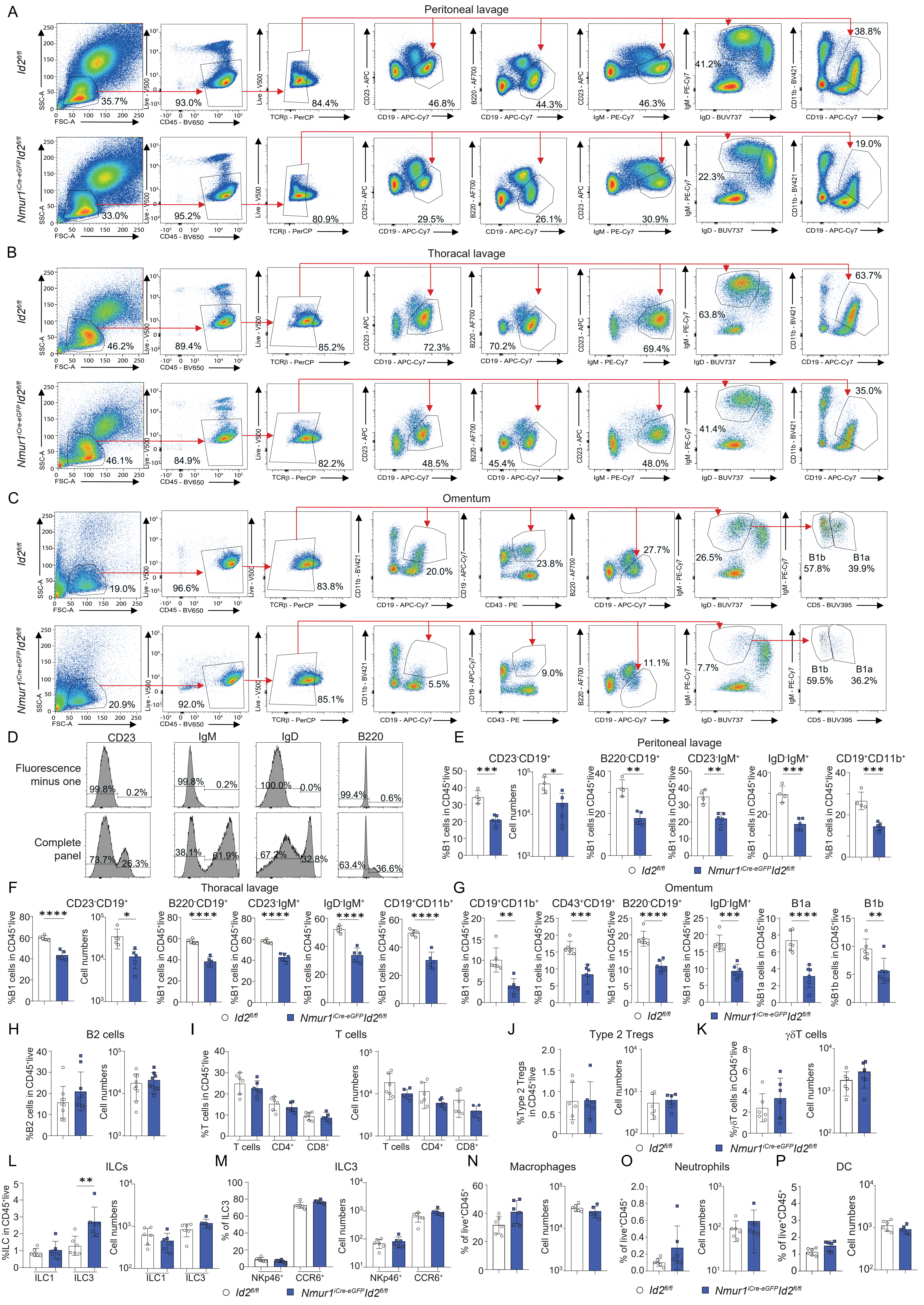
A
Peritoneal lavage
B
Thoracal lavage
C
Omentum
D
CD23
IgM
IgD
B220
Fluorescence minus one
Complete panel
E
F
Thoracal lavage
G
Omentum
H
B2 cells
I
T cells
J
Type 2 Tregs
K
γδT cells
L
ILCs
M
ILC3
N
Macrophages
O
Neutrophils
P
DC

Supplementary Figure 1 (refers to Figure 1): Different gating strategies for B1 cells show similar results and lack of ILC2s does not further alter peritoneal cellularity

a-c, Flow cytometric gating strategy of B1 cells in *Nmur1*$^{iCre\text{-}eGFP}$*Id2*$^{fl/fl}$ and littermate *Id2*$^{fl/fl}$ mice with gating via CD23- / CD19+, B220low / CD19+, CD23- / IgM+, IgDlow / IgM+, CD11b+ / CD19+ in the **a**, peritoneal cavity **b**, thoracal lavage. **c**, omentum with the adjusted gating strategy by using CD11b+ / CD19+, CD43+ / CD19+, B220low / CD19+, IgM+ / IgDlow B1 cells. B1a and B1b cells further subdivided using the marker CD5. **d**, Fluorescence minus one controls of indicated markers are shown, **e,** quantification of a (*Id2*$^{fl/fl}$ *n*=4, *Nmur1*$^{iCre\text{-}eGFP}$*Id2*$^{fl/fl}$ *n*=5), **f**, quantification of b (*Id2*$^{fl/fl}$ *n*=5, *Nmur1*$^{iCre\text{-}eGFP}$*Id2*$^{fl/fl}$ *n*=5) **g,** quantification of c (*Id2*$^{fl/fl}$ *n*=6, *Nmur1*$^{iCre\text{-}eGFP}$*Id2*$^{fl/fl}$ *n*=6). **h-p**, Flow cytometric quantification of indicated cell subsets in the peritoneal lavage in *Nmur1*$^{iCre\text{-}eGFP}$*Id2*$^{fl/fl}$ (blue bars) and littermate *Id2*$^{fl/fl}$ (white bars) mice. All cells were pre-gated on live CD45+. **h**, B2 cells were gated as TCRβ- CD23+ CD19+ (*Id2*$^{fl/fl}$ *n*=9, *Nmur1*$^{iCre\text{-}eGFP}$*Id2*$^{fl/fl}$ *n*=9). i-o (*Id2*$^{fl/fl}$ *n*=6, *Nmur1*$^{iCre\text{-}eGFP}$*Id2*$^{fl/fl}$ *n*=6). **i**, T cells were gated as CD3+ CD5+ +/- CD4+ / CD8+. **j**, Type 2 Tregs were gated as CD3+ CD4+ Foxp3+ Gata3+ KLRG1+ ST2+. k, γδT cells were gated as CD3+ CD5+ TCRγδ+. **l,m**, ILCs cells were pre-gated on Lin- (CD3 CD5 CD19 FCεR1a Ly6G) CD127+. ILC1 were further gated on NK1.1+ NKp46+. ILC3 were gated on NK1.1- c-Kit+ and subclassified in NKp46+ or CCR6+. **n**, Macrophages were gated on Lin- (CD3 CD5 CD19 FCεR1a NK1.1) CD11b+ CD64+. **o**, Neutrophils were gated on Lin- (CD3 CD5 CD19 FCεR1a NK1.1) CD11b+ CD11c+ Ly6G +. p, Dendritic cells (DC) were gated on Lin- (CD3 CD5 CD19 FCεR1a NK1.1) Ly6G- CD11b+ CD11c+. Each symbol represents data from one mouse, mean +/- SD, all data are representative of at least two independent experiments. Statistical significance was determined by two-tailed unpaired Student's t-test (e-h,j,k,n-p) or one-way ANOVA (i,l,m). *p<0.05, ** p<0.01, *** p<0.001, **** p<0.0001. PL, peritoneal lavage. Source data, including exact *p*-values, are provided as a Source data file.

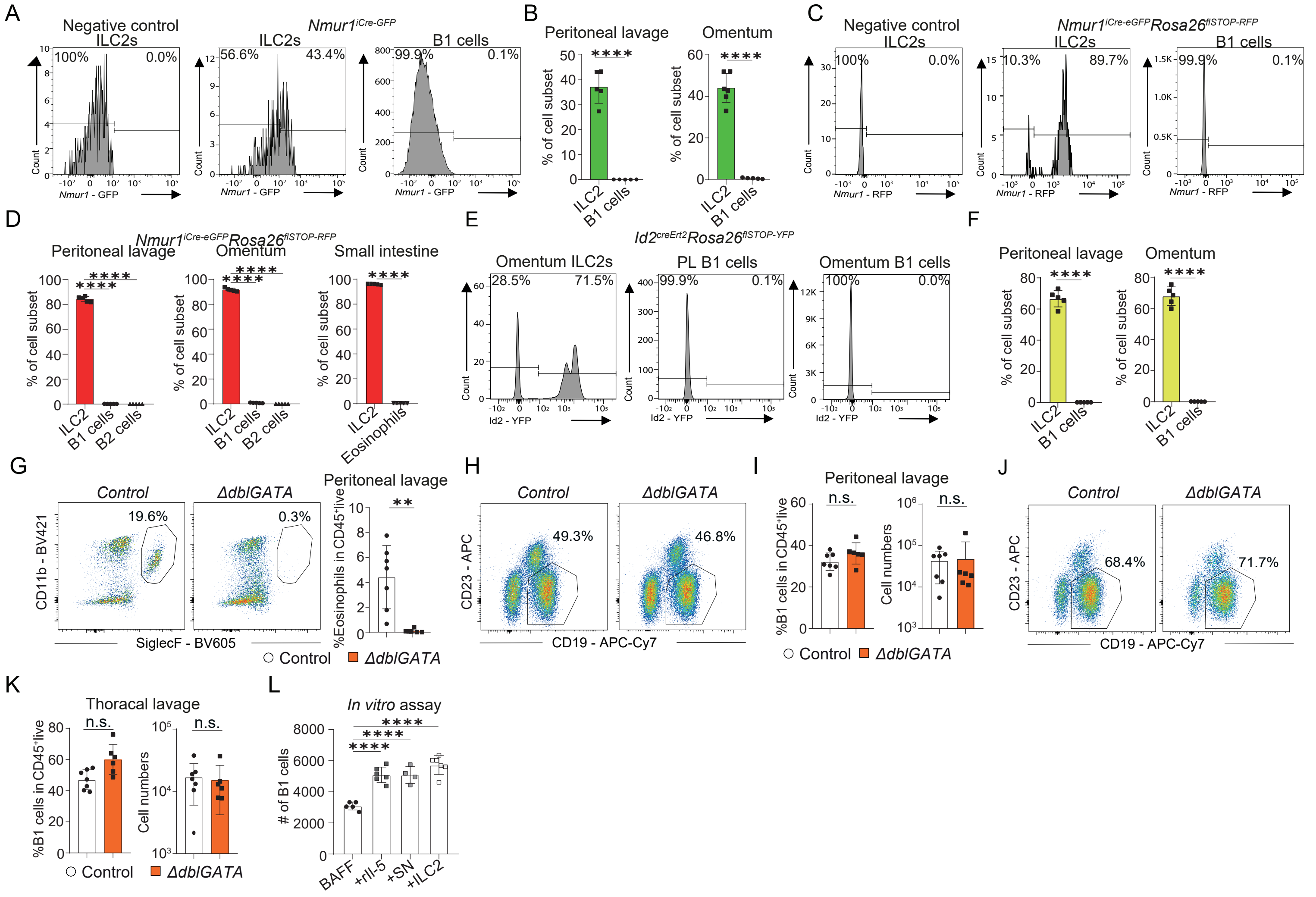

Supplementary Figure 2 (refers to Figure 1): B1 cells and eosinophils do not express Nmur1 and lack of eosinophils does not alter the B1 cell pool

a, FACS-plots and **b**, quantification of endogenous *Nmur1* (GFP) in B1 cells and ILC2s in $Nmur1^{iCre-GFP}$ mice (*n*=6). **c,d,** fate-map experiment identifying the expression of $Nmur1^{RFP}$ in B1 cells, B2 cells, eosinophils and ILC2s isolated from $Nmur1^{icre-GFP}Rosa26^{flSTOP-RFP/+}$ mice (*n*=5). **e,f,** YFP in B1 cells and ILC2s expression in $Id2^{creErt2}Rosa26^{flSTOP-YFP/+}$ mice after 5 weeks of Tamoxifen food administration (*n*=5). **g-k** Control (*4Get*) and *dblGata* (on a *4Get* background) mice were analyzed in steady state (Control *n*=7, *4Get n*=6). **g,** Flow cytometric plots and quantification of eosinophils pre-gated on live CD45+ in the peritoneal cavity. **h-k,** Flow cytometric plots and quantification of B1 cells in the **h,i,** peritoneal cavity and the **j,k,** thoracal lavage. **l,** 10^4 B1 cells (Live CD45+ CD19+ CD23-) from the peritoneal cavity were sort-purified from *n*=6 C57/BL6 wild type mice and cultured for 5 days with the addition of B-cell activating factor (BAFF). Recombinant IL-5 (rIl-5), supernatant (SN) from ILC2s pre-cultured for 3 days in vitro or 10^4 ILC2s were added. B1 cell numbers were analyzed by flow cytometry. Each symbol represents data from one mouse, mean +/- SD, all data are representative of at least two independent experiments. Statistical significance was determined by two-tailed unpaired Student's t-test (b,d,fh,i,k) or one-way ANOVA (d,l). *p<0.05, ** p<0.01, *** p<0.001, **** p<0.0001. PL, peritoneal lavage. Source data, including exact *p*-values, are provided as a Source data file.

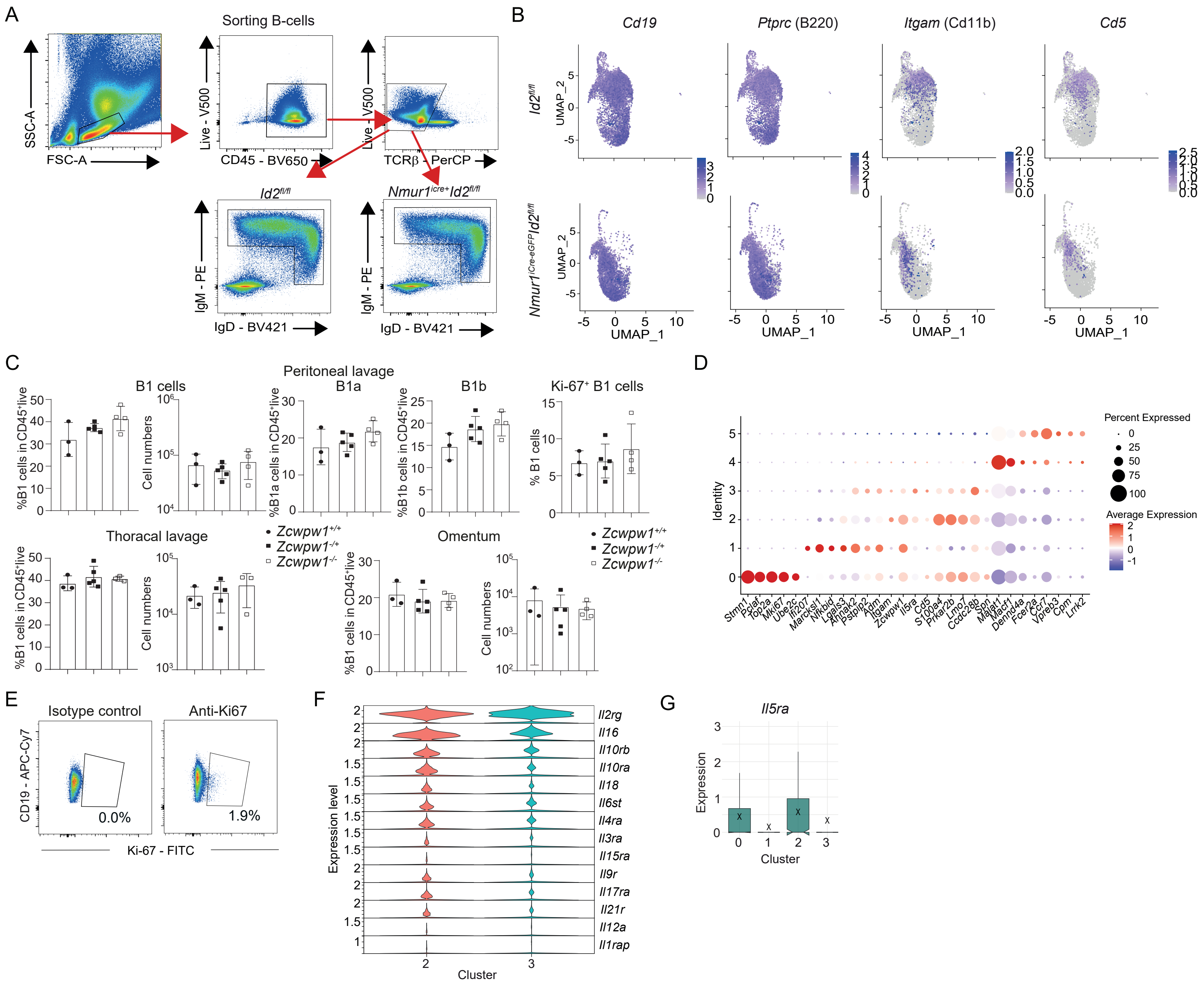

Supplementary Figure 3 (refers to Figure 2): Validation of single-cell sequencing

a, Sorting strategy of B cells from the peritoneal cavity of *Nmur1*$^{iCre\text{-}eGFP}$*Id2*$^{fl/fl}$ and littermate *Id2*$^{fl/fl}$ mice for scRNA-seq. 5 mice were pooled for the sequencing. **b,** UMAPs of general B cell surface markers (*CD19*, *Ptprc*) and B1 cell specific surface markers (*Itgam*, *Cd5*). **c,** Flow cytometric quantification of B1 cells in littermate *Zcwpw1*$^{-/-}$, *Zcwpw1*$^{+/-}$ and *Zcwpw1*$^{+/+}$ in the peritoneal cavity, thoracal lavage and the omentum at steady state (*Zcwpw1*$^{+/+}$ *n*=3, *Zcwpw1*$^{+/-}$ *n*=5, *Zcwpw1*$^{-/-}$ *n*=4). **d,** Dot-plot of the most significant genes in each Cluster from the scRNA-seq. **e,** Ki-67 of B1 cells in comparison to the appropriate isotype control. **f,** Violin plot of expressed cytokine-receptors comparing clusters 2 and 3, **g,** Boxplot of the expression of *Il5ra* in all B1 cell clusters (Clusters 0-3). Each symbol represents data from one mouse, mean +/- SD, all data are representative of at least two independent experiments. Statistical significance was determined by one-way ANOVA (c). *p<0.05, ** p<0.01, *** p<0.001, **** p<0.0001. PL, peritoneal lavage. Source data, including exact *p*-values, are provided as a Source data file.

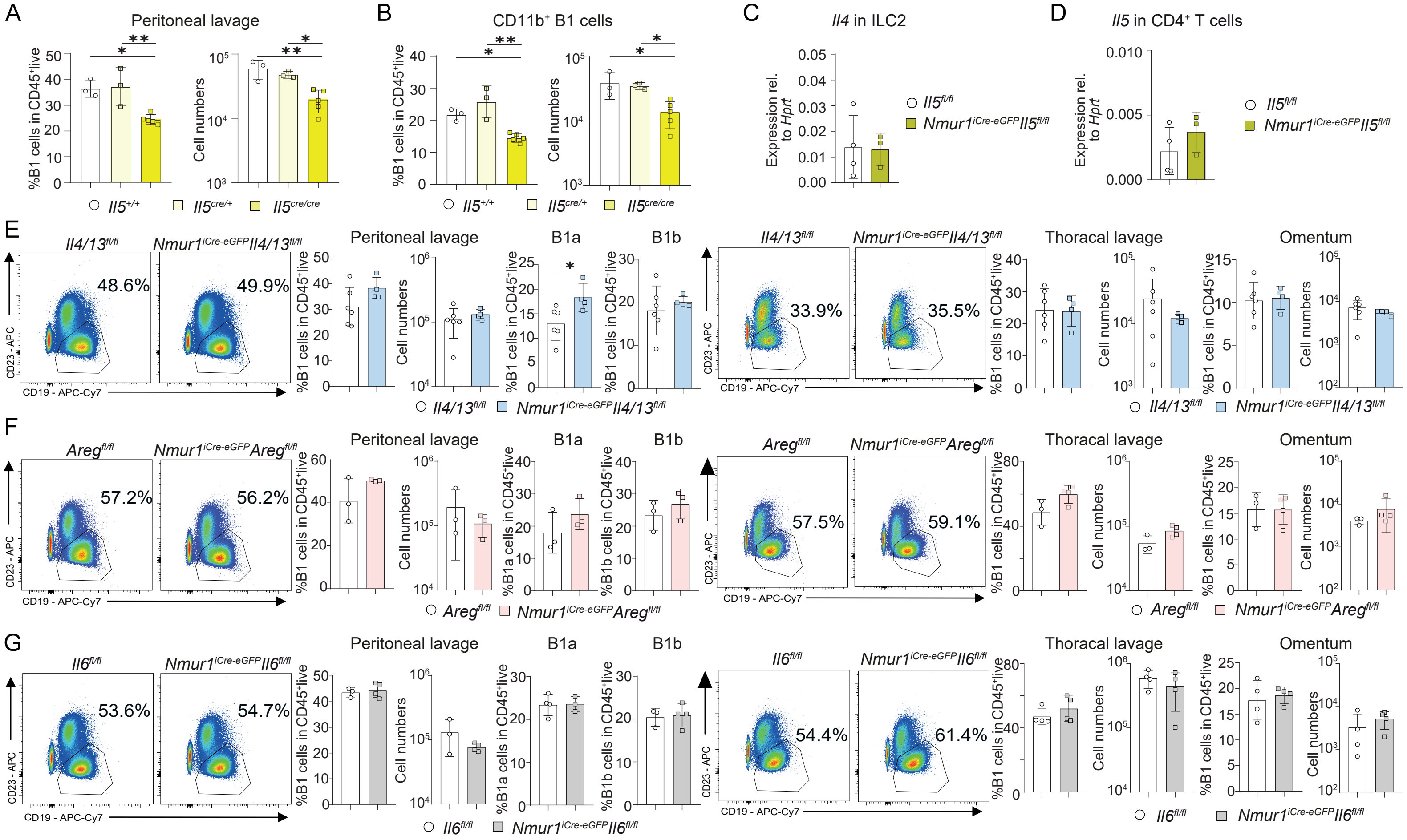

Supplementary Figure 4 (refers to Figure 3): The ILC2-derived factors IL-4/13, Areg and IL-6 do not affect B1 cell development

a,b, Flow cytometric quantification of B1 cells in *Il5*$^{+/+}$, *Il5*$^{Cre/+}$ (light yellow), and *Il5*$^{Cre/Cre}$ (yellow) from the peritoneal lavage (*Il5*$^{+/+}$ *n*=3, *Il5*$^{Cre/+}$ *n*=3, *Il5*$^{Cre/Cre}$ *n*=5). **c,d**, Validation of the newly generated mouse line *Nmur1*$^{iCre\text{-}eGFP}$*Il5*$^{fl/fl}$ by qPCR of sort-purified ILC2s gated as live CD45^{+} Lin^{-} (CD3, CD5, CD19, FcεRI, Ly6G) CD127^{+} KLRG1^{+} and CD4^{+} T cells gated as live CD45^{+} CD3^{+}, CD4^{+} from the lung (*Il5*$^{fl/fl}$ *n*=4, *Nmur1*$^{iCre\text{-}eGFP}$*Il5*$^{fl/fl}$ *n*=3). **e-g**, Flow cytometry plots and quantification of total B1, B1a and B1b cells (based on the marker CD5) of peritoneal lavage, thoracal lavage and omentum of **e**, *Nmur1*$^{iCre\text{-}eGFP}$*Il4/13*$^{fl/fl}$ and littermate *Il4/13*$^{fl/fl}$ control mice (*Il4/13*$^{fl/fl}$ *n*=6, *Nmur1*$^{iCre\text{-}eGFP}$*Il4/13*$^{fl/fl}$ *n*=4), **f**, *Nmur1*$^{iCre\text{-}eGFP}$*Areg*$^{fl/fl}$ and littermate *Areg*$^{fl/fl}$ control mice (*Areg*$^{fl/fl}$ *n*=3, *Nmur1*$^{iCre\text{-}eGFP}$*Areg*$^{fl/fl}$ *n*=4), **g**, *Nmur1*^{iCre} *Il6*$^{fl/fl}$ and littermate *Il6*$^{fl/f}$ mice (*Il6*$^{fl/f}$ *n*=4, *Nmur1*$^{iCre\text{-}eGFP}$*Il6*$^{fl/fl}$ *n*=4). Each symbol represents data from one mouse, mean +/- SD, all data are representative of at least two independent experiments. Statistical significance was determined by two-tailed unpaired Student's t-test (c-g) or one-way ANOVA (a,b). n.s. non-significant, *p<0.05, ** p<0.01. Source data, including exact *p*-values, are provided as a Source data file.

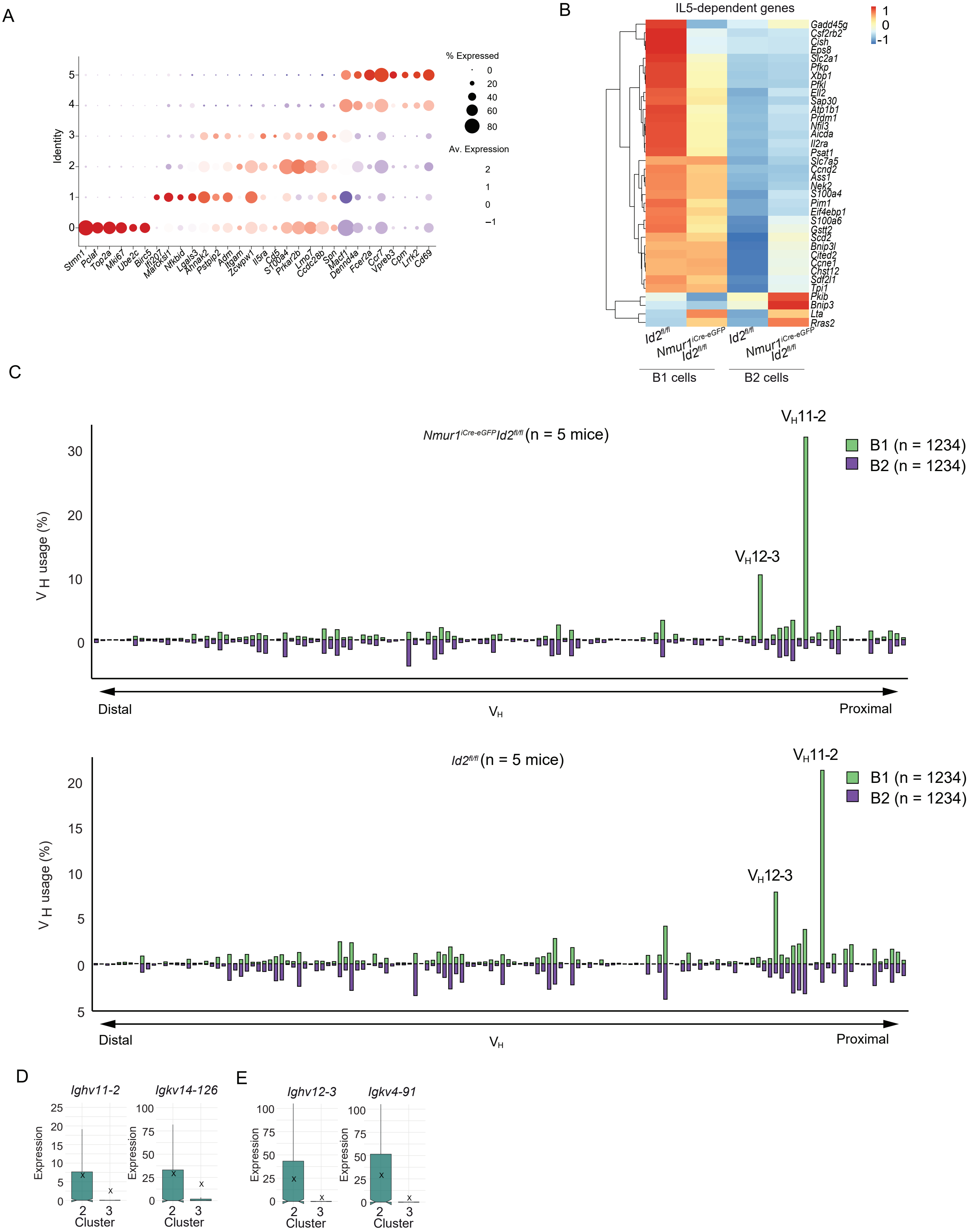

Supplementary Figure 5 (refers to Figure 4): B1 cells mainly rely on V$_H$11-2 and V$_H$12-3

a-e, Sort-purified B cells underwent single-cell sequencing including sequencing of the B-cell repertoire. Sort-purification analogous to Fig S3. 5 mice were pooled. **a**, dot-plot of the most significant genes in each Cluster excluding BCR sequencing. **b**, differentially-regulated gene expression of reported IL-5-induced B1 cell genes (21) of *Nmur1*$^{iCre\text{-}eGFP}$*Id2*$^{fl/fl}$ (blue) and littermate *Id2*$^{fl/fl}$ mice. **c**, *Ighv* usage in B1 and B2 cells from 5 mice from *Nmur1*$^{iCre\text{-}eGFP}$*Id2*$^{fl/fl}$ (upper panel) and littermate *Id2*$^{fl/fl}$ mice (lower panel). *Ighv* gene segments are ordered by their relative proximity to the D segments. Number of cells is depicted in the Figure legend. **d,e**, Quantification of the most important phosphatidyl-specific heavy- and corresponding light chains, *Ighv11-2*, *Igkv14-126,* and *Ighv12-3*, *Igkv4-91,* comparing clusters 2 and 3.

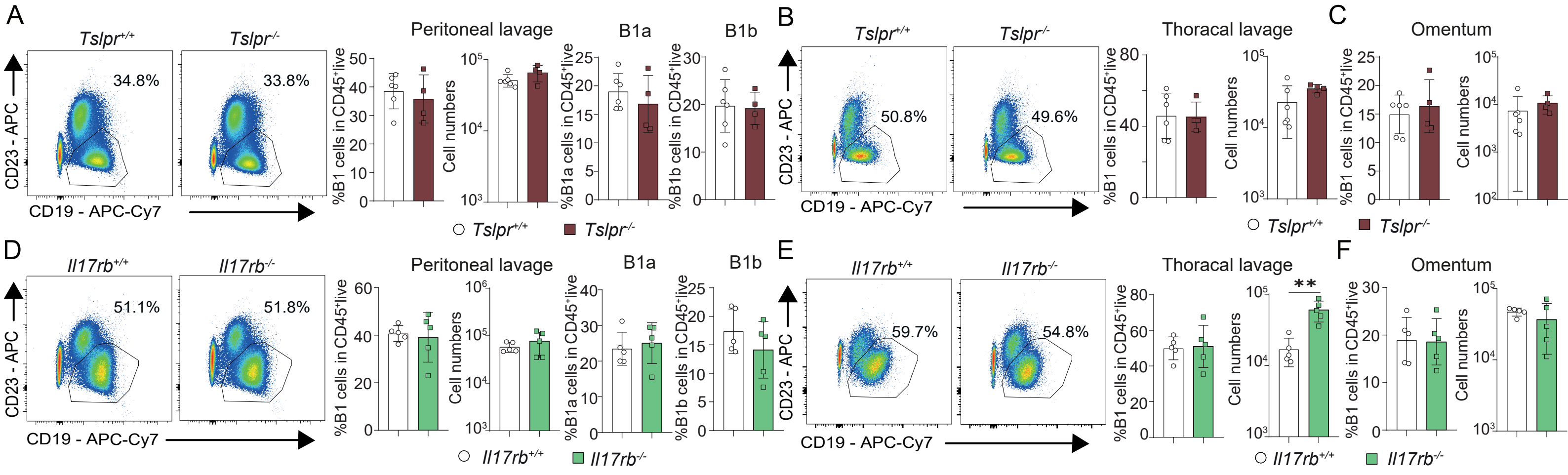

Supplementary Figure 6 (refers to Figure 6): TSLPR and IL-25 do not shape the B1 cell pool
Flow cytometry plots and quantification of total B1 cells, B1a and B1b cells of **a**, peritoneal lavage, **b**, thoracal lavage and **c**, omentum of *Tslpr*$^{+/+}$ and *Tslpr*$^{-/-}$ mice and **d**, the peritoneal lavage, **e**, thoracal lavage and **f,** omentum of *Il17rb*$^{+/+}$ and *Il17rb*$^{-/-}$ mice. Each symbol represents data from one mouse, mean +/- SD, all data are representative of at least two independent experiments. Statistical significance was determined by two-tailed unpaired Student's t-test (a-f). Source data, including exact *p*-values, are provided as a Source data file.
